# Impairment of Coronary Arteriolar Endothelium-Dependent Dilation after Multi-Walled Carbon Nanotube Inhalation: A Time-Course Study

**DOI:** 10.3390/ijms131113781

**Published:** 2012-10-24

**Authors:** Phoebe A. Stapleton, Valerie C. Minarchick, Amy M. Cumpston, Walter McKinney, Bean T. Chen, Tina M. Sager, David G. Frazer, Robert R. Mercer, James Scabilloni, Michael E. Andrew, Vincent Castranova, Timothy R. Nurkiewicz

**Affiliations:** 1 Center for Cardiovascular and Respiratory Sciences, West Virginia University School of Medicine, Morgantown, WV 26506, USA; E-Mails: pstapleton@hsc.wvu.edu (P.A.S.); vbukowsk@mix.wvu.edu (V.C.M.); 2 Department of Physiology and Pharmacology, West Virginia University School of Medicine, Morgantown, WV 26506, USA; E-Mail: dfrazer@cdc.gov; 3 Pathology and Physiology Research Branch, Health Effects Laboratory Division, National Institute for Occupational Safety and Health, Morgantown, WV 26505, USA; E-Mails: atm5@cdc.gov (A.M.C.); wdm9@cdc.gov (W.M.); bdc4@cdc.gov (B.T.C.); sst2@cdc.gov (T.M.S.); rmercer@cdc.gov (R.R.M.); jscabilloni@cdc.gov (J.S.); vic1@cdc.gov (V.C.); 4 Biostatistics and Epidemiology Branch, Health Effects Laboratory Division, National Institute for Occupational Safety and Health, Morgantown, WV 26505, USA; E-Mail: mta6@cdc.gov; 5 Department of Neurobiology and Anatomy, West Virginia University School of Medicine, Morgantown, WV 26505, USA

**Keywords:** microcirculation, coronary, arteriole, nanotoxicology, multi-walled carbon nanotube, engineered nanomaterial, translocation

## Abstract

Engineered nanomaterials have been developed for widespread applications due to many highly unique and desirable characteristics. The purpose of this study was to assess pulmonary inflammation and subepicardial arteriolar reactivity in response to multi-walled carbon nanotube (MWCNT) inhalation and evaluate the time course of vascular alterations. Rats were exposed to MWCNT aerosols producing pulmonary deposition. Pulmonary inflammation via bronchoalveolar lavage and MWCNT translocation from the lungs to systemic organs was evident 24 h post-inhalation. Coronary arterioles were evaluated 24–168 h post-exposure to determine microvascular response to changes in transmural pressure, endothelium-dependent and -independent reactivity. Myogenic responsiveness, vascular smooth muscle reactivity to nitric oxide, and α-adrenergic responses all remained intact. However, a severe impact on endothelium-dependent dilation was observed within 24 h after MWCNT inhalation, a condition which improved, but did not fully return to control after 168 h. In conclusion, results indicate that MWCNT inhalation not only leads to pulmonary inflammation and cytotoxicity at low lung burdens, but also a low level of particle translocation to systemic organs. MWCNT inhalation also leads to impairments of endothelium-dependent dilation in the coronary microcirculation within 24 h, a condition which does not fully dissipate within 168 h. The innovations within the field of nanotechnology, while exciting and novel, can only reach their full potential if toxicity is first properly assessed.

## 1. Introduction

Engineered nanomaterials (ENM), are anthropogenic materials specifically designed for their unique properties at the nanometer scale (<100 nm in one dimension) [1,2]. The uses for these materials are seemingly limitless, with an impressive amount of resources aimed at the development of engineering and biomedical applications [3]. ENM range widely with respect to physiochemical properties, including but not limited to size, shape, charge, solubility, free radical production, biological half-life, and functionalization leading to physiological responses after exposure [4]. They can be loaded, bound, or impregnated with a variety of pharmaceutical compounds, gene plasmids, or heavy metals, thereby, acting as vehicles for intentional biomedical therapeutic exposure [5]. However, ENM exposures may also occur unintentionally in the manufacturing process, as a by-product of their use, or during their breakdown [6]. Nanotechnology advancements, while exciting and novel, can only reach their full potential if the toxicity is first assessed.

Multi-walled carbon nanotubes (MWCNT) have been developed for widespread applications, including biomedical, electronic, computer, and aerospace products, due to their highly desirable mechanical, electrical, thermal, strength, and magnetic properties. The physical composition and inherent design of MWCNT allow for variation in the length or width of MWCNT, leading to an alteration of the surface area available for drug delivery, or an increase in tensile strength [7]. These spear-like carbon compounds have been shown to embed in alveolar tissue [8], producing alarmingly analogous results to asbestos exposure [9,10], as the body has considerable difficulties clearing such fibrous particles [11]. Filamentously shaped MWCNT have been demonstrated to penetrate alveolar macrophages, epithelial cells [9], pleural cells, and interstitial cells; followed closely by macrophage accumulation, an increase in oxidant production [12], the development of progressive pulmonary fibrosis, and chronic granulomatous inflammation producing a rapid and detrimental result after pulmonary exposure [13]. However, these results exclusively pertain to interactions within the pulmonary system, while the microvascular impacts remained unexamined.

Questions pertaining to the *in vivo* physiochemical properties of the MWCNT are also physiologically relevant as carbon nanomaterials have been shown to slowly translocate after pulmonary exposure to secondary target organs, a potentially hazardous outcome associated with exposure [14,15]. Studies have also been conducted to evaluate the extra-pulmonary secondary target organ effects after pulmonary exposure to MWCNT demonstrating a dose-dependent inflammation and necrosis within the liver and kidney [12], a reduction in antioxidant capacity [16], and indirect thrombogenic effects within the liver and heart [17]. Therefore, microvascular dysfunction resulting from MWCNT exposure due to translocation, secondary systemic inflammation, or an increased oxidant production associated with direct contact is plausible.

We have reported that on an equal mass basis pulmonary exposures to nano-sized titanium dioxide (TiO_2_) particles produced greater endothelium-dependent microvascular dysfunction than exposure to fine titanium dioxide particles [18]. Recent investigations in our laboratory have also determined that these endothelium-dependent effects are primarily associated with decreased nitric oxide (NO) bioavailability related to increased oxidative stress in the arteriolar wall leading to alterations in prostanoid signaling [19,20].

Our previous ENM exposure investigations have focused on TiO_2_, a spherical metal oxide, and explored its impact on systemic and coronary microvascular function. The purpose of this study was to evaluate pulmonary inflammation and subepicardial arteriolar reactivity after MWCNT inhalation, a fibrous carbon nanomaterial. A second purpose was to evaluate the time course of impairment post-inhalation. We hypothesized that MWCNT exposure would initially compromise coronary function, characterized by alterations in arteriolar reactivity, after acute exposure, and that this dysfunction would dissipate with time post-exposure.

## 2. Results and Discussion

### 2.1. Experimental Characterization

#### 2.1.1. MWCNT Aerosol Characteristics

There were no differences between the MWCNT exposure conditions that each group was exposed to with respect to aerosol concentration and calculated lung burden (Table 1). These results represent the reliability and repeatability of our inhalation exposure system, while indicating that temporal differences in microvascular reactivity were not due to differential initial dosages.

#### 2.1.2. Animal and Vessel Characteristics

There were no significant differences with respect to age, weight, heart wet weight, or mean arterial pressure (MAP) after initial MWCNT exposure or over time after exposure (Table 2). Additionally, there were no significant differences within the vessel characteristics of spontaneous arteriolar tone, maximal diameter, and active tone (Table 2). Overall, these results suggest that the basal characteristics of the rats and coronary arterioles were unaffected by MWCNT exposure.

### 2.2. Pulmonary Inflammation and Damage

**Figure 1:**
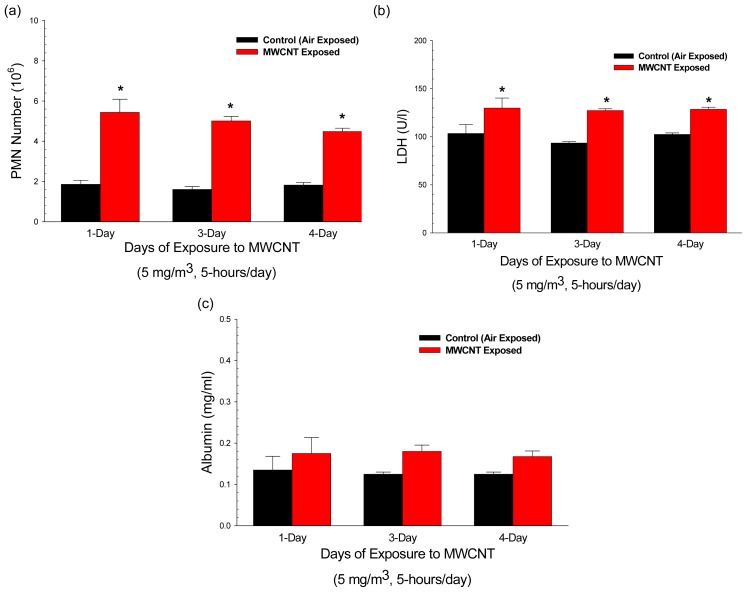
Comparison of pulmonary inflammation markers induced by inhalation exposure to 5 mg/m^3^ multi-walled carbon nanotube for 5 h per day for 1, 3, or 4 days. (**a**) Cell counts of polymorphonuclear leukocytes were used as an indicator of inflammation. Values are given as means ± SE (*n* = 8). * indicates that PMN influx for that group was significantly higher than control (*p* < 0.05). (**b**) Bronchoalveolar lavage fluid lactate dehydrogenase (LDH) was used as a marker of cytotoxicity. Values are given as means ± SE (*n* = 8). * indicates that LDH activity for that group was significantly higher than control (*p* < 0.05). (**c**) Bronchoalveolar lavage fluid albumin concentrations were used as a marker of alveolar air/blood barrier damage. Values are given as means ± SE (*n* = 8).

MWCNT inhalation caused pulmonary inflammation and damage as determined by significant increases in polymorphonuclear leukocytes (PMN) harvested by bronchoalveolar lavage (BAL) (Figure 1a) and in lactate dehydrogenase (LDH) activity (cell damage) (Figure 1b). Protein (air/blood barrier damage) (Figure 1c) in BAL fluid exhibited non-significant increases of 40% after MWCNT exposure. Pulmonary responses remained relatively constant at lung burdens from 13.5 to 54.1 ug/lung. These data indicate that pulmonary inflammation and cellular damage occurred within 24 h of MWCNT inhalation even at a low lung burden of 13.5 μg/lung.

Mouse aspiration studies from our group reported that MWCNT causes acute lung inflammation and damage at lung burdens of 10–80 μg/mouse. In addition, granulomatous lesions and interstitial fibrosis which persisted for at least 56-days were reported at lung burdens of 20–80 μg/mouse [8,9,21]. This study is similar to previous results indicating pulmonary inflammation and cytotoxicity at low lung burdens of ENM exposure [22–25].

In comparison to rat verses human exposures, the equivalent human lung dose is approximately 3.4 mg [26]. This dosage is not considered substantial, as burdens in the human lung considered pathological are in the multiple gram range for known toxicants (silica, coal dust, *etc*.).

When comparing pulmonary inflammatory responses across several particle types (fine TiO_2_[27], fine ROFA [27], ultrafine TiO_2_[22], or MWCNT) at dosages ranging from 13.5 μg/rat in this study to 250 μg/rat fine ROFA examined by this group, the potency sequence was MWCNT > ultrafine TiO_2_ > fine ROFA = fine TiO_2_. Therefore, MWCNT evaluated in this study lead to the greatest pulmonary inflammatory outcome at the lowest toxicological dosage.

### 2.3. Multi-Walled Carbon Nanotube Translocation

**Figure 2:**
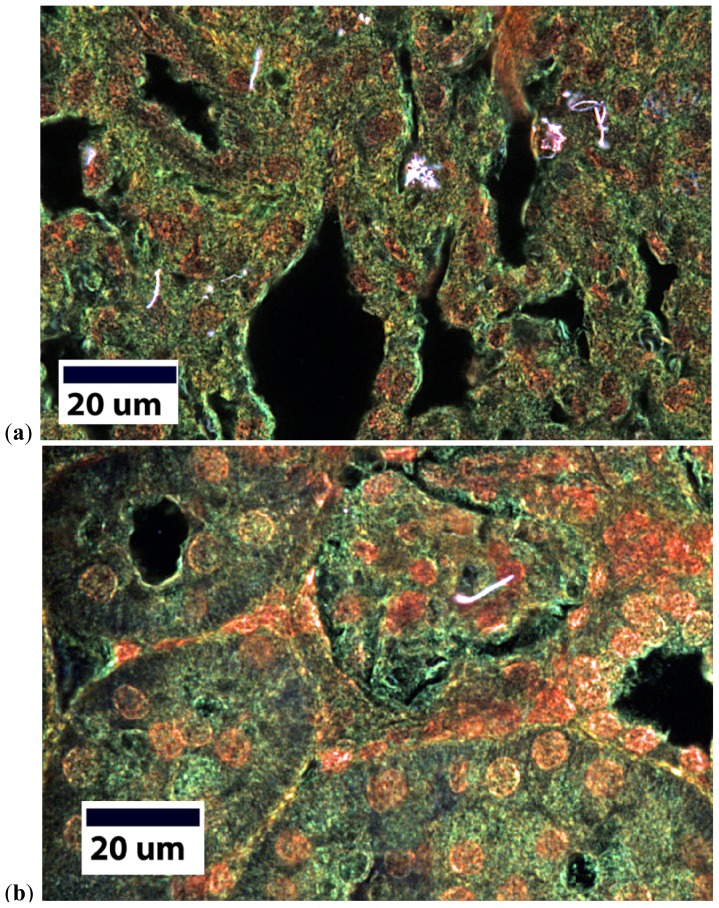
Enhanced darkfield imaging of MWCNT within the lung (**a**) and the kidney (**b**). MWCNT appear under enhanced darkfield as bright white structures.

Enhanced darkfield imaging of lung sections readily demonstrated MWCNT in lung sections as illustrated in Figure 2a. Deposited MWCNT structures in the lungs ranged from singlet fibers to larger structures containing many CNT. MWCNT were also identified in the three systemic organs (kidney, liver, heart) examined. In all cases the extra-thoracic MWCNT were single fibers, 3–6 microns in length. Figure 2b shows an example of a MWCNT in the kidney.

Transport of MWCNT to systemic organs expressed in terms of MWCNT fibers and in terms of a percentage of total lung burden for the kidney, liver, and heart are given in Table 3. The liver, which is also the largest organ of the three examined, had the largest number of fibers; the heart, the smallest organ, had the fewest fibers. Translocation of MWCNT from the lung to each systemic organ was a small fraction of one percent over the 24 h post-exposure period.

MWCNT have been shown to translocate from the lungs after inhalation or intratracheal instillation, in this report and others, indicating the possibility for direct systemic vascular interaction [12]. In this study, we found a small number of MWCNT within the liver, kidney, heart 24 h after pulmonary exposure. It is reasonable to expect to find some translocated MWCNT in the kidney, a clearance organ, in preparation to eliminate the ENM. MWCNT found within the liver may not be representative of the number throughout systemic organs, as the liver is a larger sequestering organ. Given the heterogeneity in organ function, size, and anatomy a true count of MWCNT number may prove not only difficult, but misleading. For example, the impact of MWCNT within the heart would be very different than those found in the liver and kidney, as coronary function is based on cellular signaling, pathways, and interactions all operating in unison. Therefore, a single MWCNT piercing the small delicate concert of the impulse-generating cells could contribute to significant untoward consequences. The concept of direct interaction due to translocation is not unique to MWCNT, as other materials after pulmonary exposure have also been found systemically [15,29–32].

Translocation from the lung is not the only avenue for direct ENM contact. Other intentional non-pulmonary routes of exposure (intravenous injection for biomedical drug delivery or imaging) must be considered. This direct interaction may play a role in alterations to endothelium-dependent signaling patterns, as endothelial cells may be injured due to intimate contact [8], heightened inflammatory response [33], induction of ENM-driven NO scavenging [34], and an increase in reactive species (oxidative or nitrosative) [35].

MWCNT have been shown to produce a direct biological effect both *in vitro* and *in vivo.* In co-culture, endothelial and epithelial cells have shown an increase in pro-inflammatory signaling and cytokine production (including NF-κB, IL-6, and IL-8), an enhancement of MAP kinase phosphorylation, a disruption of cellular cytoskeleton, severe functional impairments, and a reduction in cellular viability [10,36–38]. Walk *et al*. [37] also reported that these endothelial outcomes may be dependent on the MWCNT dose. Furthermore, *in vivo* MWCNT exposure, at higher concentrations than those described within this study, has led to other outcomes independent of pulmonary outcomes including increases in hepatic platelet adhesion, fibrinogen deposition, increase in vascular permeability, systemic inflammation, and decreased anti-oxidant capabilities [16,17,39]. The decrease in anti-oxidant enzyme activity could lead to an increase in oxidant stress and subsequent NO scavenging [16]. Additionally, increases in platelet adhesion may induce thromboxane A2 generation, thereby creating a vasoconstrictive environment [17].

### 2.4. Coronary Isolated Microvessel Reactivity

#### 2.4.1. Endothelium-Dependent Responses

**Figure 3:**
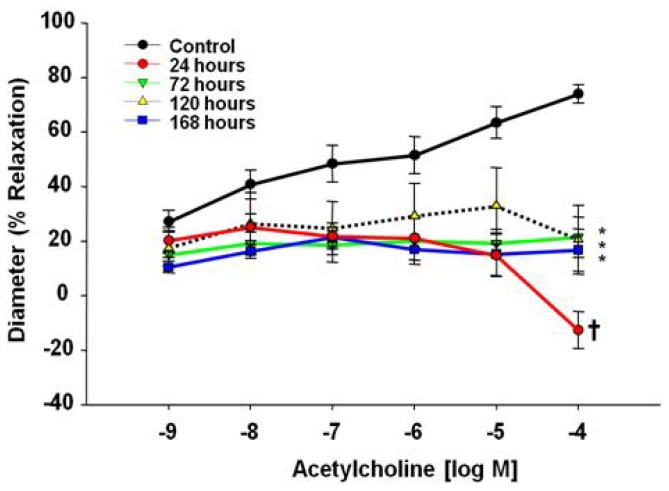
Acetylcholine time-course dose response curve at 24–168 h after control (*n* = 8 rats) or MWCNT inhalation (*n* = 7–14 rats). Values are mean ± SE. *p < 0.05 *vs.* Control. **†***p* < 0.05 for 24 h compared to all other groups. MWCNT inhalation attenuates coronary arteriolar endothelium-dependent dilation.

Endothelium-dependent dilator responses were significantly attenuated in subepicardial arterioles following pulmonary exposure to MWCNT. This impairment was evident 24 h post-exposure (7% at 10^−9^ M ACh to 87% at 10^−4^ M ACh from control) and remained blunted through 168 h post-exposure (17% at 10^−9^ M ACh to 57% at 10^−4^ M ACh from control) (Figure 3). Vascular reactivity to ACh was impaired to such a degree that vasoconstriction ensued at the highest ACh dose (1 × 10^−4^), indicating a shift in mechanistic signaling.

**Figure 4:**
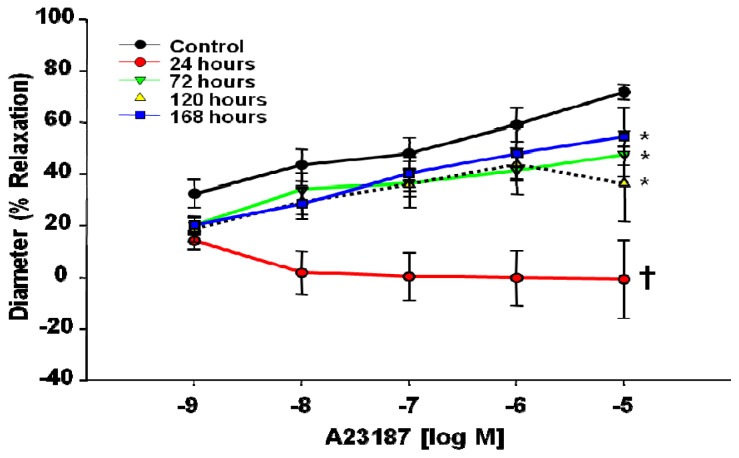
A23187 time-course dose response curve 24–168 h after control (*n* = 6 rats) or MWCNT inhalation (*n* = 6–12 rats). Values are mean ± SE. *p < 0.05 *vs.* Control. **†** indicates *p* < 0.05 24 h compared to all other groups. Time course of impaired endothelium-dependent dilation in coronary arterioles after MWCNT inhalation.

With respect to the Ca^2+^ ionophore A23187, endothelium-dependent reactivity was similarly blunted throughout the dosages (1 × 10^−9^–1 × 10^−5^ M) at the 24 h time point (18% at 10^−9^ M A23187 to 73% at 10^−5^ M A23187 from control) (Figure 4). These results indicate that MWCNT inhalation impairs endothelium-dependent dilation 24 h post-exposure. Although this impairment dissipated over time, normal reactivity did not fully return to control levels even after 168 h post-inhalation (12% at 10^−9^ M A23187 to 17% at 10^−4^ M A23187 from control).

Endothelium-dependent dilation was examined via two pathways: ACh and A23187 Ca^2+^ signaling. Differences in either pathway may be attributed to a decrease in nitric oxide bioavailability, due to reduced production or oxidative scavenging of NO. While, A23187 operates via an increase in intracellular Ca^2+^ for signal transduction, the ACh results may indicate acute shifts in NO bioavailability in combination with compensatory arachidonic acid metabolites (prostacyclin, thromboxane A2, lipoxygenase) leading to the lack of reactivity exhibited at the 24 h time point. Regardless of the agonist, this poor endothelial reactivity may be indicative of competing vasoactive metabolites, a decrease in dilation due to NO bioavailability, or an increase in the potent vasoconstrictor thromboxane A2. Collectively, this may lead to an acute blunting of endothelium-dependent dilation 24 h after MWCNT exposure. As time passes post-exposure, endothelium-dependent dilation slowly improves with respect to both ACh and A23187 responses, but neither regained full reactivity back to control levels within 168 h post exposure. These signaling alterations can be seen in a number of cardiovascular disease states, but have only recently been described in response to ENM exposure [18,19,40–44].

The results described in this manuscript differ from past microvascular results to TiO_2_ exposure. Both ENM exposures impaired endothelium-dependent dilation within 24 h; however, TiO_2_inhalation attenuated dilation to ACh and A23187, while MWCNT inhalation leads to a constriction in response to ACh and a blunted response to A23187 [18,19,41]. These results indicate inhalation of either nanomaterial (TiO_2_ or MWCNT) will impair endothelium-dependent dilation; however, the pathway and severity of said impairment is unique and specific to each ENM. In these early studies, it appears that both TiO_2_ and MWCNT impact endothelium-dependent reactivity associated with NO signaling, indicated by the impairment of dilation in response to A23187. MWCNT inhalation may also impact arachidonic acid signaling through ACh dose response, possibly to a greater degree than TiO_2_ inhalation, as evident by the constriction at higher doses of ACh not seen after TiO_2_ inhalation. Interestingly, when comparing microvascular endothelium-dependent dilation inhibition across several particle types and dosages investigated by our group, the potency sequence is very similar to that for inflammation, *i.e.*, MWCNT > ultrafine TiO_2_ > fine ROFA = fine TiO_2_[20,24]. Therefore, MWCNT induced the greatest pulmonary inflammation and the greatest inhibition of endothelium-dependent dilation.

#### 2.4.2. Vascular Smooth Muscle Responsiveness

**Figure 5:**
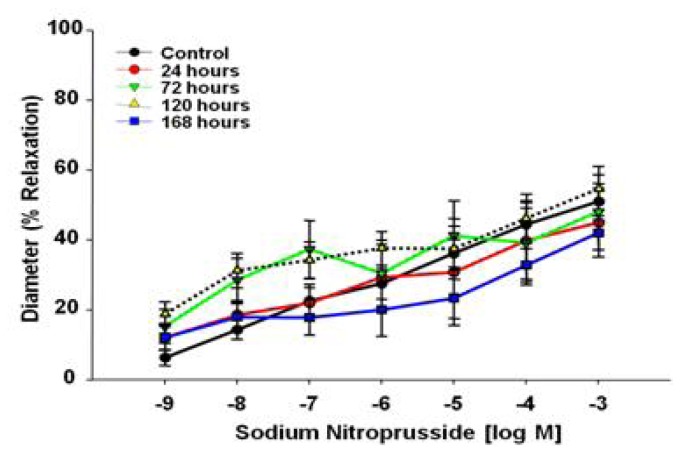
Sodium nitroprusside time-course dose response curve 24–168 h after control (*n* = 8 rats) or MWCNT inhalation (*n* = 7–8 rats).Values are mean ± SE. MWCNT inhalation does not impair microvascular reactivity of smooth muscle to NO signaling.

Sodium nitroprusside (SNP) was used as an NO donor to evaluate endothelium-independent vasodilation. There were no significant differences between the groups, indicating that MWCNT exposure does not alter vascular smooth muscle NO sensitivity in the coronary microcirculation (Figure 5).

**Figure 6:**
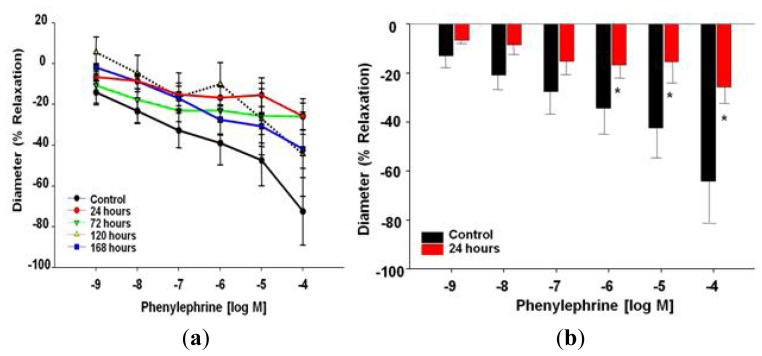
(**a**) Phenylephrine time-course dose response curve 24–168 h after control (*n* = 8 rats) or MWCNT inhalation (*n* = 7–9 rats). Values are mean ± SE. Time course evaluation does not reveal any significant differences between control and MWCNT exposed rats. (**b**) Responsiveness to phenylephrine of control (*n* = 8 rats) *vs.* MWCNT (24 h post-inhalation; *n* = 7 rats) coronary arteriolar smooth muscle. Values are mean ± SE. There are significant differences between control and rats exposed to MWCNT via inhalation after 24 h at the highest concentrations of PE.

Phenylephrine (PE) was used to evaluate vascular smooth muscle α-adrenergic sensitivity. There were no significant differences associated with coronary arteriolar constriction, with respect to time, due to MWCNT exposure (Figure 6a), *i.e.*, the dose response curve between these groups was not significantly different (*p* = 0.08). However, statistical significance was obtained after further point-by-point analysis comparing individual doses of PE between the control and 24 h group, at the three highest dosages of PE (10^−6^–10^−4^ M) (Figure 6b). These results indicate a possible alteration of α-adrenergic responsiveness to high dosages of PE 24 h after MWCNT inhalation.

Responses to PE, when the highest dosages were compared independently between the control and 24 h time point, were statistically significant (Figure 6b). When analyzing this outcome, there are four points that must be considered, this may be an avenue of additional exploration. First, what is the biological relevance or physiological impact to coronary blood flow regulation at these dosages? To consider this point, one should keep in mind the distribution of α-1-adrenergic receptors within the coronary microcirculation [45,46]. Second, is it physiologically meaningful to compare these PE dosages or are these supraphysiological dosages useful to further investigate mechanistic outcomes? Third, is this a true physiological difference 24 h after MWCNT inhalation? Lastly, could this be a protective compensatory mechanism associated with significant endothelial-dependent dysfunction to maintain coronary perfusion? This could indeed be a protective mechanism, as arachidonic acid signaling is possibly shifted toward greater thromboxane production leading to an overall constrictive environment; perfusion is maintained by dampening other constrictive signals.

#### 2.4.3. Active Pressure Response

**Figure 7:**
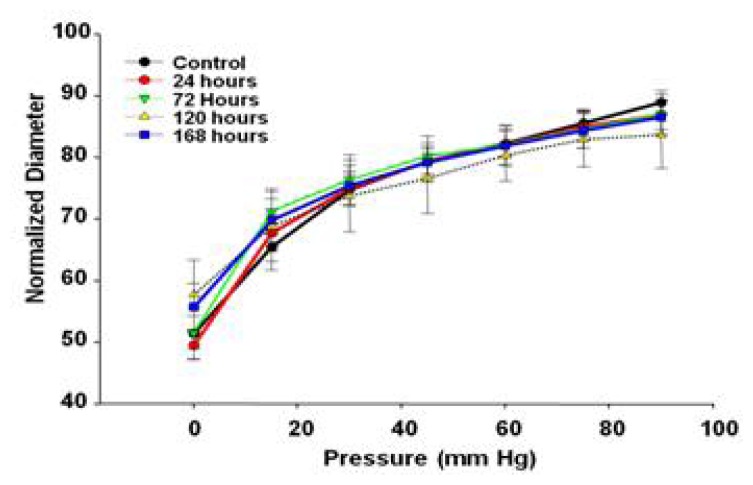
Myogenic responses of coronary arterioles 24- to168 h after control (*n* = 10 rats) or MWCNT inhalation (*n* = 7–13 rats). Values are mean ± SE. Data indicate no significant differences to active pressure responses after MWCNT inhalation.

There were no significant differences in the myogenic responsiveness to pressure alterations between 0 and 90 mm Hg (Figure 7), indicating that the sub-epicardial arterioles are able to respond appropriately to changes in arteriolar transmural pressure after MWCNT inhalation.

Myogenic responsiveness was evaluated as a contributor to tissue autoregulation. The inability for a microvessel to accurately conduct and transduce force could have dire tissue perfusion consequences. Altered myogenic regulation has been shown in many pathological states, including hypertension, which may ultimately lead to an increase in total peripheral perfusion [47]. This responsiveness and autoregulatory assessment could not have been accurately attained if the vessels were not permitted to develop spontaneous tone and instead were preconstricted above physiological norms. In this study, myogenic responsiveness was maintained at all time points over changes in transmural pressure, indicating compensatory mechanisms may be activated to maintain autoregulation, despite endothelial dysfunction.

### 2.5. Schematic Representation

**Figure 8:**
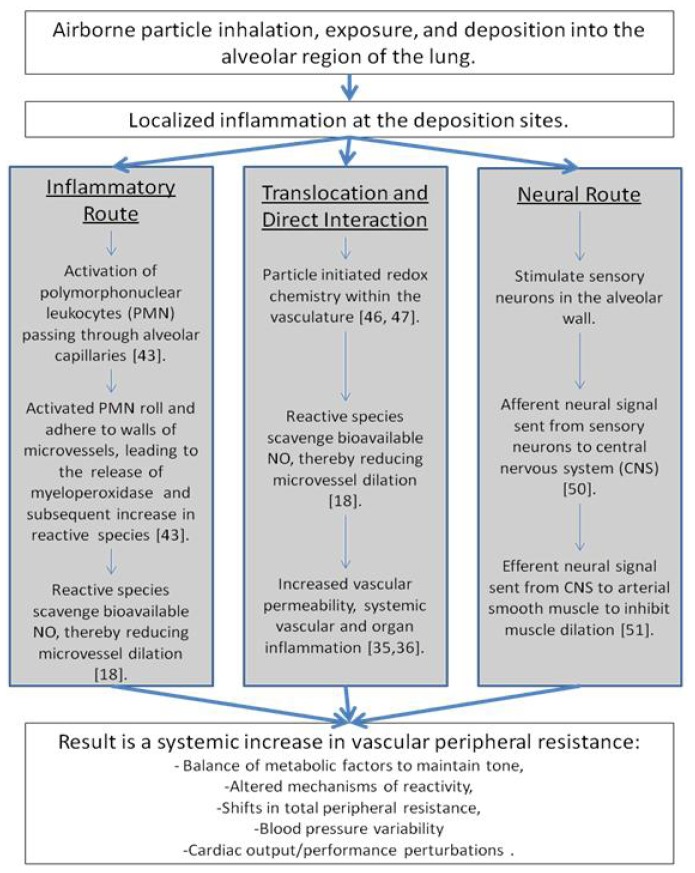
Schematic representation of mechanistic alterations initiated by xenobiotic pulmonary exposure leading to cardiovascular impairments. The key point of this schematic is that no single pathway or mechanism leads to the cardiovascular outcomes described within the literature. Additionally, interactions between the pathways may exist and account for the temporal maintenance of these consequences. Furthermore, consequences in terms of morbidity or mortality are not likely to be seen in young, healthy populations. Rather, these events may be contributing to or exacerbate a pre-existing condition.

Overall, our group has developed a schematic representing the mechanistic alterations leading from pulmonary exposure to cardiovascular implications that may contribute to morbidity and mortality rates (Figure 8). In this depiction, a pulmonary exposure stimulates bronchoalveolar inflammation [21,22,25,48,49].

At this point, there are three routes which may influence cardiovascular function, leading to increases in morbidity and mortality rates [50]. First, the inflammatory route hypothesizes an activation of PMN, leading to the release of myeloperoxidase (MPO) and an increase in reactive species, which scavenge bioavailable NO [20,48]. Secondly, translocation or direct interaction may initiate redox chemistry within the vasculature, as these ENM may be manufactured from or coated with a variety of highly reactive materials capable of producing reactive species or reducing antioxidant competence within a physiological environment [16,51,52]. Lastly, the neural route may stimulate sensory neurons leading to altered afferent and efferent signaling patterns ultimately effecting the maintenance of smooth muscle tone [53,54]. These pathways are not isolated or independent of each other and they may operate in conjunction with the other routes. For example, a translocated particle may embed in tissue (as evident in Figure 2), causing a localized inflammation and influencing the inflammatory route, leading to the same systemic vascular outcome.

These cardiovascular results may not manifest severely in the young healthy models traditionally evaluated; however, in compromised models of cardiovascular disease, such as diabetes, metabolic syndrome, hypertension, angina, and/or hypercholesterolemia, these exposures may intensify or worsen pre-existing conditions. Combining these exposures and pre-existing conditions with an increase in physical activity, as during exercise or occupational activity, and the possibility exists for the development of a public health storm leading to increases in morbidity and mortality rates described in epidemiological literature [55–58].

## 3. Experimental Section

### 3.1. Experimental Animals

Specific pathogen free male Sprague Dawley [Hla:(SD)CVF] rats (7–8 weeks old) were purchased from Hilltop Laboratories (Scottsdale, PA, USA). Rats were housed in an AAALAC approved animal facility at the National Institute for Occupational Safety and Health in laminar flow cages under controlled temperature and humidity conditions and a 12 h light/dark cycle and acclimated for 5 days before use. The animals were monitored to be free of endogenous viral pathogens, parasites, mycoplasms, Helicobacter and CAR Bacillus. Animals were housed in ventilated cages which were provided HEPA-filtered air, with Alpha-Dri virgin cellulose chips and hardwood Beta-chips used as bedding. The rats were maintained on a ProLaB 3500 diet and tap water, both of which were provided *ad libitum*. To ensure that all methods were performed humanely and with regard to alleviation of suffering, all experimental procedures were approved by the Institutional Animal Care and Use Committees of the West Virginia University and the National Institute for Occupational Safety and Health.

### 3.2. Engineered Nanomaterials

The MWCNT material was provided by Mitsui & Co. (MWNT-7, Lot # 061220-31, Ibaraki, Japan) and has been previously characterized by our group [21,28]. The nanotubes were catalytically grown by chemical vapor deposition processes. The powder was conductive and contained nanomaterials of fiber-like shape containing an extremely high purity (>99.5%) of carbon with a trace amount of iron. The average width of the primary particles was between 40 and 90 nm (mean = 49 nm), while the length varied and could be up to several micrometers (mean = 3.9 μm). The specific surface area was 24–28 m^2^/g.

### 3.3. Inhalation Exposure

**Figure 9:**
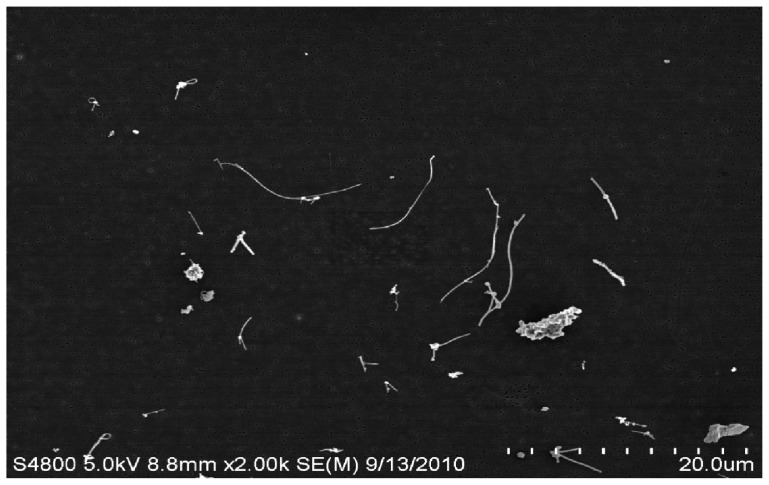
Electron microscopy image of aerosolized MWCNT.

**Figure 10:**
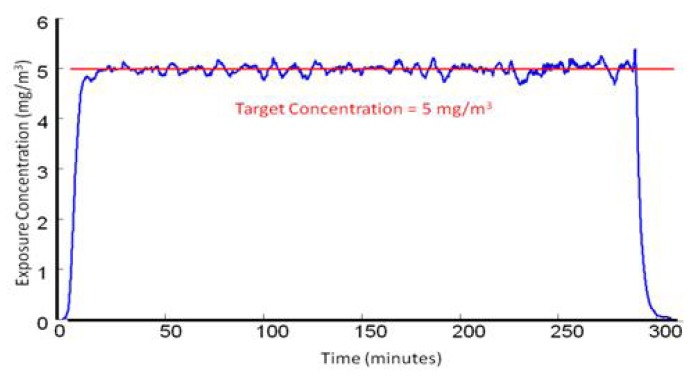
MWCNT aerosol generation maintained at 5 mg/m^3^ for the 5 h exposure.

We have previously reported and described the MWCNT aerosol generator exposure system used for inhalation exposures in the current experiments [59]. Briefly, the inhalation system was developed for small rodent inhalation particulate exposure. It consists of an acoustic generator, an animal housing chamber, many monitoring devices, and feed-back control. The MWCNT aerosol generator includes a large acrylic cylindrical chamber enclosed by flexible latex rubber diaphragms. The MWCNT were placed on the lower diaphragm, inside the chamber. The chamber was mounted vertically above a high compliance loudspeaker facing upwards, acoustically coupling the chamber and speaker. The speaker was computer operated (LabVIEW 7.1, National Instruments: Austin, TX, USA) using an analog signal through an audio amplifier (Butt Kicker, model BKA-1000-4A) which varied between 10 and 18 Hz over a 20-s period allowing aerosolization of the MWCNT from the diaphragm. The aerosol within the rat exposure chamber was continuously monitored with a Data RAM to allow real time feedback concentration control. Clean dry air entered low into the chamber allowing the smallest of these aerosolized ENM to exit through a tube at the top of cylinder, while larger agglomerates remained in the lower portion. These well dispersed aerosolized MWCNT (Figure 9) were directed to an animal exposure chamber at 5 mg/m^3^ (Figure 10), while a variety of instruments were used to characterize the aerosol: a Data RAM (DR-40000 Thermo Electron Co, Franklin, MA, USA) for monitoring the mass concentration in real time, a combined APS/SMPS device (TSI, Shoreview, MN, USA) for monitoring the particle size distribution of the aerosol, a MOUDI (MSP, Shoreview, MN, MN, USA) for measuring mass-based aerodynamic size distribution, and two air filters to provide gravimetric measurements. In addition, temperature, relative humidity, pressure and airflow rate in the chamber were monitored, controlled and recorded by a computer throughout the 5 h exposure.

Rats were exposed (5 mg/m^3^, 5 h/day) for 1, 3, or 4 days to obtain three different lung burdens (13.5, 40.1, and 54.1 μg/lung) for pulmonary dose response studies. These lung burdens were determined based on mouse methodology previously reported by our group [49] and normalized to rat minute ventilation. Coronary microvascular response was initially evaluated at these three lung burdens. However, microvascular data reported in this manuscript were at the lowest lung burden (13.5 μg/lung) as microvascular inhibition was attained at this low dosage. After inhalation exposures, all rats recovered for at least 24 h prior to experimental procedures.

### 3.4. Pulmonary Inflammation

#### 3.4.1. Bronchoalveolar Lavage

At 1-day post-exposure, the animals were euthanized with an i.p. injection of sodium pentobarbital (>100 mg/kg body weight) and exsanguinated by cutting the descending aorta. A tracheal cannula was inserted and BAL was conducted [60]. The first lavage wash contained a 6 ml aliquot of cold Ca^+2^ and Mg^+2^-free phosphate buffered saline (PBS), which was flushed into and out of the lungs twice before the lavage fluid was collected. After the initial lavage wash, the BAL continued with 8 mL aliquots of cold Ca^+2^ and Mg^+2^-free PBS until an additional 80 mL of bronchoalveolar lavage fluid (BALF) was collected. The BALF was then centrifuged at 600 × *g* for 10 min using a Sorvall RC 3B Plus centrifuge (Sorvall Thermo Electron Corporation, Asheville, NC, USA). After centrifugation, the supernatant from the first lavage wash was decanted into a clean conical vial and was stored on ice to be used for cytotoxicity analysis. The supernatant from the subsequent lavage washes was discarded. The pelleted cells from all lavages were washed with cold Ca^+2^ and Mg^+2^-free PBS and respun at 600 × *g* for 10 min after which the supernatant was discarded and the cells were resuspended in 1 mL of HEPES buffer.

#### 3.4.2. Cell Counts and Differentials

BAL cell counts were conducted according to their unique cell diameters, using an electronic cell counter (Beckman Coulter Multisizer 3 Counter, Hialeah, FL, USA). Cytospin preparations of the BAL cells were made using a cytocentrifuge (Shandon Elliot Cytocentrifuge, London, UK). The cytospin preparations were stained with modified Wright-Giemsa stain, and cell differentials we determined by light microscopy [21].

#### 3.4.3. BAL Fluid Lactate Dehydrogenase Activity and Albumin Concentration

The degree of cytotoxicity induced by the inhaled MWCNT was determined by lactate dehydrogenase (LDH) activity in the BAL fluid from the initial wash. LDH activity was measured using Roche COBAS MIRA Plus chemical analyzer (Roche Diagnostic Systems Inc., Branchburg, NJ, USA). The alveolar air/blood barrier damage was determined by the concentration of albumin in the BAL fluid. BAL fluid albumin concentrations were measured using a CobasFara II Analyzer (Roche Diagnostic Systems, Montclair, NJ, USA) as previously described [60].

### 3.5. Enhanced-Darkfield Light Microscopy Imaging of Nanoparticles

Following sacrifice, organs were removed, sliced into 2–3 mm thick tissue blocks and fixed by immersion. Tissue blocks were prepared for lung, heart, kidney, and liver. After overnight fixation, tissue blocks were embedded in paraffin and sectioned at 5 micron thickness. Sections were collected on ultrasonically cleaned, laser cut slides (Schott North America, Inc., Elmsford, NY, USA) to avoid nanoparticle contamination from the ground edges of traditional slides. To enhance contrast between tissue and MWCNT, sections were stained with Sirius Red. Sirius Red staining consists of immersion of the slides in 0.1% Picrosirius solution (100 mg of Sirius Red F3BA in 100 mL of saturated aqueous picric acid, pH 2) for 1 h followed by washing for 1 min in 0.01 M HCl. Sections were the briefly counterstained in freshly filtered Mayer’s hematoxylin for 2 min, dehydrated, and coverslipped.

The optical system used for enhanced-darkfield imaging consisted of high signal-to-noise, darkfield-based illumination optics adapted to an Olympus BX-41 microscope (CytoViva, Auburn, AL, USA). After alignment of the substage oil immersion optics with a 10× objective, the entire area of each section was scanned at 20× to detect any MWCNT. Any potential MWCNT identified at low power were confirmed by examination at 100× oil immersion objective. Enhanced darkfield images were taken with a 2048 × 2048 pixel digital camera (Dage-MTI Excel digital camera XLMCT, Michigan City, IN, USA). Additional details on preparation and imaging of MWCNT in tissue sections have previously been described [9].

Counts of the number of MWCNT fibers identified in each section and the cross-sectional area were tabulated for each tissue block. The number of MWCNT per unit volume was determined by dividing the counts of fiber number by the volume (tissue area × section thickness) of each block used in counting. The results were expressed as number of MWCNT in each organ determined by multiplying the number of MWCNT per unit volumes times the specific organ volume.

### 3.6. Isolated and Perfused Sub-Epicardial Microvessel Preparation

Rats were anesthetized with isoflurane (5% induction, 2% maintenance) and the heart was removed, flushed of excess blood, and placed in a dish of chilled (4 °C) physiological salt solution (PSS) at 24-, 72-, 120-, or 168 h post-inhalation as previously described by our laboratory [19,41]. Briefly, coronary resistance arterioles (<160 μm maximum diameter) were isolated from the left anterior descending (LAD) artery distribution, transferred to a vessel chamber (Living Systems Instrumentation, Burlington, VT, USA) containing fresh oxygenated PSS, cannulated with glass pipettes, and secured using nylon suture (11-0 ophthalmic, Alcon, UK). Arterioles were extended to their *in situ* length, pressurized to 45 mm Hg with physiological salt solution (PSS), superfused with warmed (37 °C) oxygenated PSS at a rate of 10 mL/min, and allowed to develop spontaneous tone [41,61]. Vessel diameters were measured using video calipers (Colorado Video, Boulder, CO, USA).

Following equilibration, arteriolar reactivity was randomly evaluated to ensure that responses were neither interactive nor time-dependent in response to: (1) pressure changes to elicit a myogenic response, *i.e.*, reductions and increases in pressure from 0 mm Hg to 90 mm Hg at 15 mm Hg increments; (2) acetylcholine (ACh) (10^−9^–10^−4^ M); (3) sodium nitroprusside (SNP) (10^−9^–10^−3^ M); (4) phenylephrine (PE) (10^−9^–10^−4^ M); and (5) the calcium ionophore, A23187 (10^−9^–10^−5^ M). Following assessments of arteriolar reactivity, the superfusate was replaced with Ca^2+^-free PSS until passive tone could be established.

### 3.7. Formulae and Statistical Analysis

Data are expressed as means ± SE. Spontaneous tone was calculated by the following equation: [(D_M_ − D_I_)/D_M_] × 100, where D_M_ is the maximal diameter recorded at 45 mm Hg under Ca^2+^-free PSS as described above, and D_I_ is the initial steady-state diameter achieved prior to experimental period. Vessels were used for experiments only if spontaneous tone ≥20% was achieved. Active responses to pressure changes were normalized to the maximum diameter as previously described: Normalized Diameter = D_SS_/D_M_, where D_SS_ is the steady-state diameter maintained at each pressure phase [41]. The experimental responses to ACh, A23187, SNP, and PE are presented as percent of relaxation from baseline diameter: [(D_SS_ − D_CON_)/(D_M_ − D_CON_)] × 100, where D_SS_ remains the steady-state diameter achieved after each chemical bolus, and D_CON_ is the control diameter measured immediately prior to the dose-response experiment. All experimental periods were at least 2 min in duration, and all steady-state diameters were collected for at least 1 min. Representing the responses in this manner allowed us to normalize for potential differences in baseline diameters before each dose-response curve.

Statistical comparisons were made at 24 h post-inhalation (5 mg/m^3^, 5 h) for dose response values of percent maximum dilation from treatment of vessels with ACh, A23187, myogenic reactivity, phenylephrine, and SNP between air and MWCNT-exposed groups. These comparisons were made using a repeated measures analysis of variance (ANOVA) approach with SAS PROC MIXED (SAS). This analysis approach allows for the correct specification of a within subject’s correlation structure and also accommodates unequal variances between CONTROL and MWCNT treatments. The repeated effect was within animal over dose of ACh *etc.*, and an autoregressive correlation structure over increasing dose was found to best fit these data. The ANOVA model was based on a two way treatment structure with nanoparticle (CONTROL *vs.* MWCNT) as the first factor and dose of ACh etc. as the second factor. The overall test for significance was for a treatment by dose interaction followed by post –hoc comparisons at each dose level.

Statistical comparisons were also made for the linear part of each dose response curve using a random coefficient model that allows estimation of a dose response slope for each subject and provides an aggregate comparison of the dose response slopes between treatments at each follow-up time. This allowed for comparison of the mean linear dose slopes between CONTROL and MWCNT for each follow-up time.

## 4. Conclusions

There are five key findings in this study. First, MWCNT inhalation can acutely cause pulmonary inflammation at lung burdens as low as 13.5 μg/lung within a rat model (Figure 1). Secondly, MWCNT can translocate at a very low rate out of the lungs and be visualized within other organ systems (liver, kidney, and heart) 24 h after inhalation exposure (Figure 2). Third, MWCNT inhalation leads to profound alterations to sub-epicardial endothelium-dependent dilation within 24 h (Figures 3 and 4). Although this dysfunction partially dissipated over time, it did not fully return to baseline levels within 168 h. Fourth, endothelium-independent responses to SNP (Figure 5) and PE (Figure 6a), which evaluate vascular smooth muscle sensitivity to nitric oxide signaling and α-adrenergic signaling respectively, remained unaltered after MWCNT exposure with respect to time, indicating that the coronary microvascular smooth muscle NO and adrenergic sensitivities were intact and reactive. Lastly, mechanotransduction (myogenic responsiveness) also remains intact (Figure 7). Collectively, these results are the first to report coronary microvascular dysfunction after MWCNT inhalation, a condition which persists at least 168 h after exposure.

There are many avenues which remain for further exploration: (1) evaluation of the underlying mechanisms leading to endothelium dysfunction after MWCNT exposure; (2) identification of the time point of greatest dysfunction (may be less than 24 h) and a return to baseline (past 168 h); (3) examination of the microvascular effects of other routes of MWCNT exposure (injection, gavage), assessing gender differences associated with ENM exposure, and4) evaluation of epigenetic concerns associated with maternal ENM exposure. Lastly, since diverse ENM are produced at a rate faster than their toxicological assessments can be made, high throughput predictive screening tests should be explored.

## Figures and Tables

**Table 1:** Exposure and dosage characteristics of control and multi-walled carbon nanotube (MWCNT) exposed rats.

Time Post Inhalation	*N*	Aerosol Concentration × Time ((mg/m^3^) × h)	Lung Burden (μg/lung)
Control	11	0	0
24 h	16	23 ± 1	12.6 ± 1.4
72 h	8	26 ± 1	14.3 ± 1.6
120 h	9	23 ± 1	12.6 ± 1.4
168 h	12	24 ± 1	13.1 ± 1.4

**Table 2:** Animal and arteriolar characteristics of control and MWCNT exposed rats.

Time Post Inhalation	N	Age (weeks)	Weight (g)	MAP (mm Hg)	Heart Wet Weight (g)	Active Diameter (μm)	Passive Diameter (μm)	Active Tone (%)
Control	11	8.4 *±* 0.2	350 ± 4	111 ± 7	1.17 ± 0.03	96 ± 7	128 ± 7	25 ± 2
24 h	16	8.7 *±* 0.2	343 ± 4	105 ± 3	1.12 ± 0.02	100 ± 4	131 ± 5	23 ± 2
72 h	9	8.7 ± 0.2	349 ± 4	105 ± 4	1.18 ± 0.03	109 ± 6	141 ± 4	23 ± 3
120 h	10	9.1 ± 0.1	364 ± 3	108 ± 6	1.16 ± 0.03	97 ± 7	129 ± 7	25 ± 4
168 h	12	9.2 ± 0.1	357 ± 12	112 ± 2	1.17 ± 0.02	105 ± 7	136 ± 5	24 ± 3

**Table 3:** Systemic MWCNT translocation from the lungs 24 h after inhalation exposure.

Systemic Distribution of Inhaled Multi-Walled Carbon Nanotubes 1		
Organ	# of MWCNT Fibers 2	% Lung Burden
Lung	646.8 × 10^6^ *	99.99890
Kidney	1533 ± 530	0.00024
Liver	4535 ± 1100	0.00070
Heart	525 ± 1260 †	0.00008

1 Total lung burden of MWCNT following inhalation was 13.2 μg;

2 Conversion from weight of MWCNT to number of MWCNT was based on a value of 49 million MWCNT fibers per microgram [28]. Data are Mean ± SE for an N of 6 animals.

* or † indicates *p* < 0.05 *vs.* other organs.
